# Kinetically controlled self-assembly of Zn-porphyrin nanostructures *via* surfactant-assisted micelle formation

**DOI:** 10.1039/d5ra09001f

**Published:** 2025-12-09

**Authors:** Yun Mi Park, Ka Young Kim, Juyeong Kim, Ji Ha Lee, Jong Hwa Jung, Sung Ho Jung

**Affiliations:** a Department of Chemistry, Gyeongsang National University Jinju 52828 Republic of Korea shjung@gnu.ac.kr; b Technical Support Center for Chemical Industry, Korean Research Institute of Chemical Technology (KRICT) 45 Jongga-ro, Jung-gu Ulsan 44412 Republic of Korea; c Institute of Advanced Chemistry, Gyeongsang National University Jinju 52828 Republic of Korea; d Institute for Fiber Engineering and Science (IFES), Interdisciplinary Cluster for Cutting Edge Research (ICCER), Shinshu University Ueda 386-8567 Japan

## Abstract

In this study, we explore the self-assembly of zinc *meso*-tetra (4-pyridyl) porphyrin (Zn-TPyP) into nanotubes *via* a surfactant-assisted micelle formation strategy under kinetic control. Incorporating a chiral surfactant bearing alkyl chains and carboxylic acid groups proved essential for stabilizing micelle formation, thereby suppressing spontaneous assembly into thermodynamically favored structures. This encapsulation-induced micelle formation enables the kinetically controlled nucleation and anisotropic growth of Zn-TPyP nanotubes. Notably, the resulting nanotubes exhibited photocatalytic activity by effectively degrading methyl orange (MO) under visible light irradiation. Our study provides mechanistic insight into kinetic control of self-assembly processes and demonstrates the potential of micelle encapsulation as a versatile tool for engineering functional metallo-supramolecular materials with tailored functional properties.

## Abstract 2

Zn-TPyP nanotubes were formed *via* surfactant-assisted micelle encapsulation, enabling kinetically controlled self-assembly. The resulting J-aggregates showed enhanced optical and photocatalytic properties.

## Graphical abstract

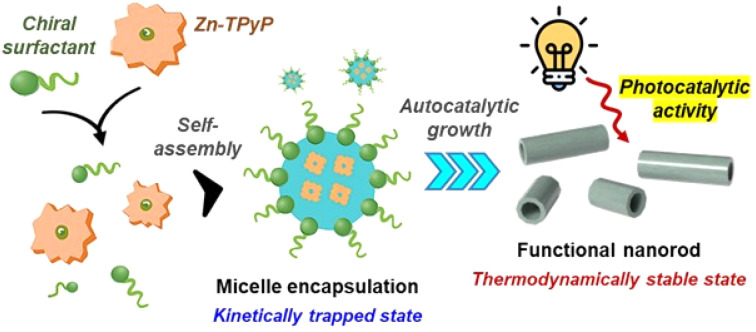

## Introduction

Molecular self-assembly is one of the rotational strategies for synthesizing hierarchical nanomaterials, which exhibit multifunctional properties derived from individual molecular building blocks.^1^ By controlling the aggregation of small molecules through non-covalent intermolecular interactions, it is possible to form supramolecular structures that mimic natural systems.^2^ This approach not only enables the creation of responsive supramolecular architectures but also offers significant potential for applications due to their stimuli-responsiveness and electronic properties.^3^ The unique properties of molecular assemblies are determined not only by the size, shape, and composition of the molecular building blocks but also by the ordered spatial arrangement within the assembly. Despite recent advances in assembling molecular building blocks into well-defined superstructures, the synthesis of hierarchical structures that utilize the structural advantages of individual molecules remains a significant challenge.

Supramolecular artificial structures consisting of porphyrin and related building blocks can be influenced and regulated by factors such as concentration, pH, ionic strength, temperature, and the presence of surfactants in bulk or at solid/liquid interfaces.^4^ The ability to control the self-assembly of porphyrins into well-defined nanostructures is crucial for tuning their optoelectronic properties and enhancing their functional performance, such as light harvesting and photocatalytic systems, as well as supramolecular polymerization.^5^ Specifically, the self-assembly behaviour of metalloporphyrins, such as zinc *meso*-tetra (4-pyridyl) porphyrins (Zn-TPyP), has been extensively studied due to their unique photophysical characteristics and structural versatility.^4*d*,5^ However, despite extensive research, the kinetically regulated self-assembly of Zn-TPyP, particularly at liquid/liquid interfaces, remains unexplored.^4*c*,6^

Recent developments in supramolecular chemistry have emphasized the importance of kinetic control over dynamic structural and functional states during the self-assembly process to achieve complexity and advanced functionalities.^1,6,7^ Creating a temporally programmable non-equilibrium state within a supramolecular system offers an effective approach for facilitating dynamic self-assembly in both living and transient supramolecular polymerizations. For instance, the exploitation of metastable states arising from spontaneous self-assembly pathways suppressed by competing pathways and intramolecular hydrogen bonding has proven useful for directing the dynamic behaviour of self-assembly in these systems.^7,8^ Moreover, strategies driven by chemical fuels, utilizing chemical or enzymatic reactions to achieve transient states, have been investigated.^7*c*,9^ These strategies allow the reversible switching between activated and dormant monomer states, influenced by chemical modifications during assembly and disassembly processes. However, strategies that exploit encapsulation to suppress spontaneous self-assembly are relatively rare.^10^ Fan and co-workers reported a surfactant-assisted cooperative self-assembly strategy to achieve precisely controlled shape, size, and composition of nanostructures.^5*b*,*e*,11^ Building on this insight, we investigate a micelle-encapsulation method that kinetically traps Zn-TPyP monomers, delaying their aggregation into nanotubes.

**Scheme 1:**
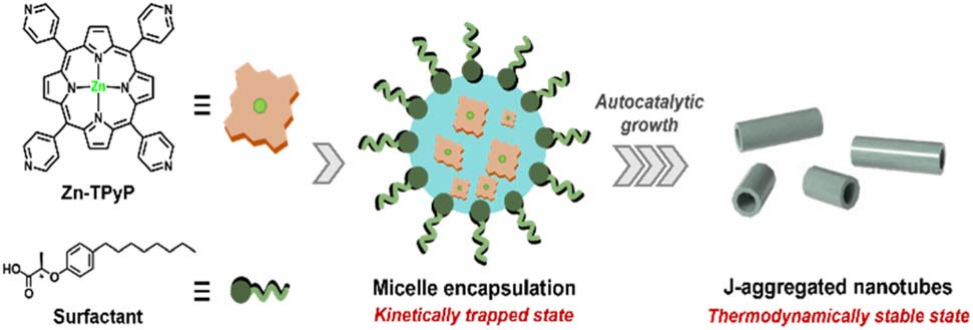
Schematic representation of the self-assembly pathway of Zn-TPyP with surfactant, from kinetically trapped micelle formation into the thermodynamically stable nanotube state.

Herein, we present a surfactant-assisted approach for the kinetic control of Zn-TPyP self-assembly. A chiral surfactant bearing alkyl chains and terminal carboxylic acid groups is employed to form micelles that encapsulate Zn-TPyP, enhancing kinetic stability and suppressing thermodynamic aggregation (Scheme 1). This strategy enables kinetically controlled nucleation and anisotropic growth of nanotubes through coordination disruption and confinement effects. Our results contribute to a deeper understanding of kinetic control in metallo-supramolecular systems and pave the way for functional nanomaterial design.

## Results and discussion

To investigate the encapsulation of Zn-TPyP by surfactant in a kinetically controlled self-assembly process, we selected a Zn-porphyrin derivative having four pyridine groups at the outer core, which can undergo axial coordination bonds and π–π stacking interactions.^12^ A chiral surfactant containing alkyl chains and a terminal carboxylic acid group was synthesized to facilitate the encapsulation process for Zn-TPyP. The synthetic details and characterization data are described in the supplementary information.

**Fig. 1:**
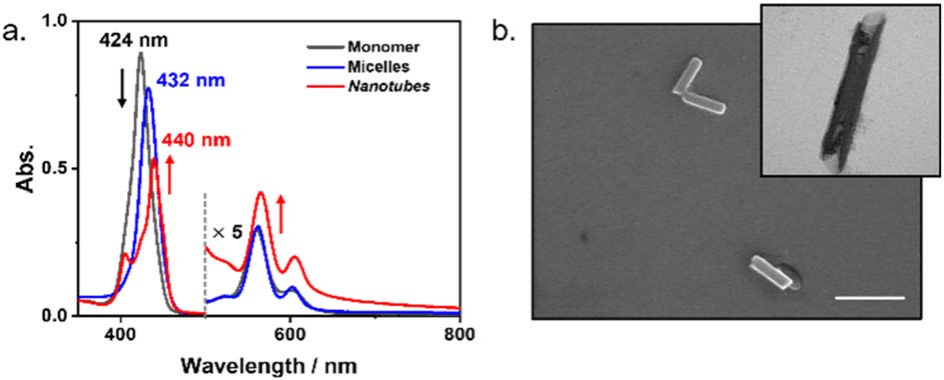
(a) UV-vis absorption spectral changes of Zn-TPyP (50 µM) in the presence of surfactant (0.9 mM). (b) SEM and TEM images of J-aggregated Zn-TPyP nanotubes (scale bar = 500 nm).

In the initial experiment, the self-assembly processes of Zn-TPyP with surfactant were observed using UV-vis absorption spectroscopy, scanning electron microscopy (SEM), transmission electron microscopy (TEM), and optical imaging. To initiate self-assembly in the aqueous phase, the pyridine groups of Zn-TPyP were acidified at 0.01 M HCl, resulting in the formation of the soluble tetrapyridinium cation Zn-TPyP-H_4_^4+^.^13^ This species exhibited a typical monomeric Soret band at 424 nm and weak Q-bands between 500 and 800 nm in the absorption spectrum (Fig. 1). A basic surfactant aqueous solution was prepared with a surfactant concentration above the critical micelle concentration (CMC, Fig. S1). The self-assembly process was initiated by injecting the Zn-TPyP-H_4_^4+^ acid solution into the basic surfactant solution under vigorous stirring, in which the final pH value is *ca.* 12.5 as an optimized condition. As shown in Fig. 1a, a significantly different absorption spectrum in the Soret band from monomeric Zn-TPyP-H_4_^4+^ was observed over time, indicating a rearrangement of the Zn-porphyrin core *via* acid-base neutralization in self-assembly. The initially formed Zn-TPyP shows a red-shifted Soret band at 432 nm. SEM observation further confirmed the formation of nanoparticles, suggesting that micelle formation led to the Zn-TPyP nanoparticles (Fig. S2).

Interestingly, after a lag time, a split Soret band at 440 nm appeared in time-dependent absorption spectra changes, indicating the formation of short-slipped J-aggregates in the porphyrin stacking mode.^14^ Furthermore, the Q-bands of the J-aggregate at 565 and 605 nm are enhanced in absorption intensities compared with those of the initially formed nanoparticles and monomeric species at 561 and 602 nm, respectively. SEM observation revealed the morphology of Zn-TPyP nanotubes with a diameter of *ca.* 50 nm, and TEM confirmed their uniform electron contrast in nanotubes (Fig. 1b and S3). In addition, photoluminescence measurements of the Zn-TPyP nanotubes reveal two emission peaks centred at 613 and 660 nm, which exhibit a smaller Stokes shift than monomeric Zn-TPyP. This is characteristic of J-aggregates (Fig. S3).

To elucidate the formation and growth mechanism of the Zn-TPyP nanotubes, we investigated the evolution of micelles-assisted cooperative self-assembly under kinetically controlled condition. Initially, Zn-TPyP was protonated into Zn-TPyP-H_4_^4+^, which was encapsulated in surfactant micelles. The acid-base neutralization deprotonates the tetra-pyridinium cations in the self-assembly process, while the interesting transformation of initial micelles of Zn-TPyP with surfactants into the J-aggregated nanotubes was observed *in situ* using UV-vis spectroscopy (Fig. 1a). The time-dependent changes in absorption spectra at 298 K clearly confirm that the spontaneous nucleation of Zn-TPyP was retarded by the encapsulated formation of porphyrin within the hydrophobic interiors of the micelles. Therefore, we suggest that the Zn-TPyP nanoparticles are kinetically trapped in micelle formation. Interestingly, the micelle formation of Zn-TPyP is completely transformed into thermodynamically favoured Zn-TPyP nanotubes after a lag time, resulting in the encapsulated formation of porphyrin within approximately 20 hours.

**Fig. 2:**
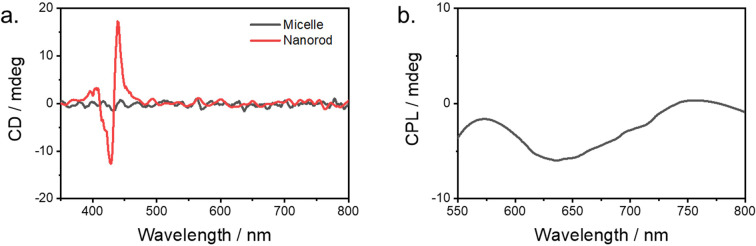
(a) CD spectra of Zn-TPyP in micelle and nanotube formation. (b) CPL spectrum of Zn-TPyP nanotubes.

Additionally, the induced weak cotton effect of Zn-TPyP nanotubes was observed in the CD spectrum, as compared with micelle formation, suggesting that the chirality of chiral surfactant is transferred into porphyrin nanotubes (Fig. 2). As a control, no CD signal was observed when an achiral surfactant (sodium dodecylbenzene sulfonate, SDBS) was used under identical conditions (Fig. S4), confirming that the chirality originates from the chiral surfactant. More interestingly, the Zn-TPyP nanotubes exhibited circular polarized luminescence (CPL) signal in the fluorescence range. The *g*_lum_ value of the negative CPL signal was 2 × 10^−4^ (Fig. S6). This observation further supports the encapsulated formation of porphyrin formed by a chiral surfactant.

**Fig. 3:**
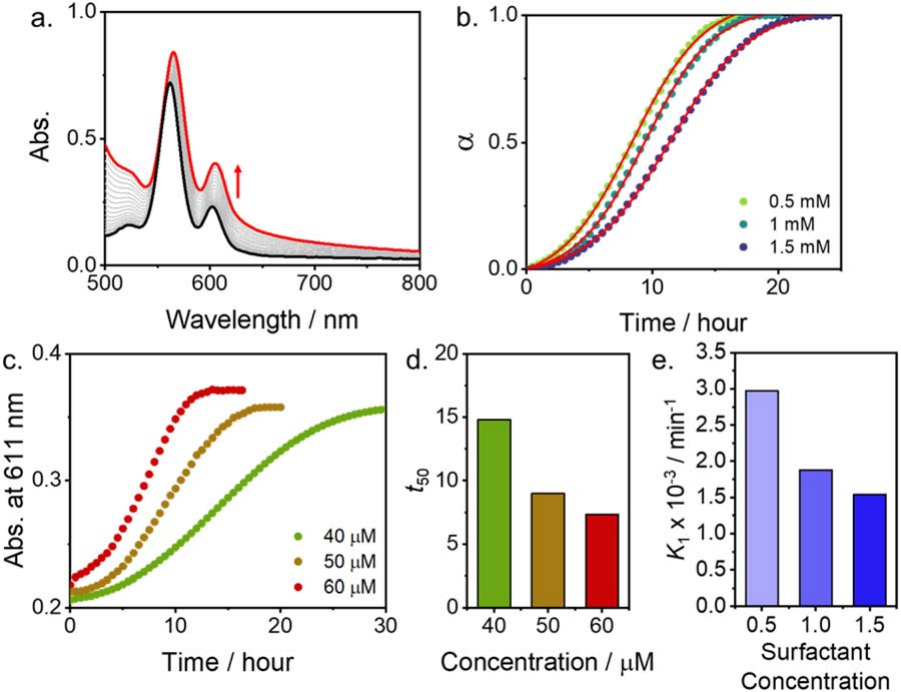
(a) Time-dependent UV-vis absorption spectra changes of Zn-TPyP (50 µM) with surfactant (0.9 mM). (b) Time-dependent degree of aggregation of Zn-TPyP (50 µM) at different surfactant concentrations. (c) Plot of absorption at 611 nm as a function of time at different Zn-TPyP concentrations (40–60 µM) with surfactant (0.9 mM). (d) Dependences of Zn-TPyP concentrations on the lag time for transformation from micelle formation into J-aggregated nanoroad. (e) Dependences of surfactant concentrations on the nucleation rate (*k*_1_) for transformation from micelle into J-aggregated nanotube.

In the kinetic experiment, during the lag phase, Zn-TPyP remained in nanoparticles due to stable micelle formation at the initial stage. Upon applying ultrasonication for 10–60 seconds at 293 K, the growth time for Zn-TPyP nanotubes was reduced, indicating that ultrasonication accelerates the nucleation process. This acceleration bypasses the lag phase and directly initiates the growth step, leading to the formation of Zn-TPyP nanotubes (Fig. S7). This result supports the formation of Zn-TPyP nanotubes followed by nucleation and growth, thus enabling kinetic control over these processes. The observed sigmoidal kinetic curve is characteristic of autocatalytic processes. For further insights, the kinetic growth was fitted using the Finke-Watzky (F–W) two-step model.^15^ The rate constants *k*_1_ and *k*_2_ for nucleation and autocatalytic growth of Zn-TPyP nanotubes (50 µM) with surfactant (1.0 mM) were estimated to be 1.8 × 10^−4^ min^−1^ and 5.1 × 10^−3^ µM^−1^ min^−1^, respectively (Fig. 3, S8, and Table S1).

The concentration-dependent changes in absorption spectra at 611 nm of Zn-TPyP indicate that the transformation process is faster when the total concentration increases from 40 µM to 60 µM (Fig. 3c and d). These kinetic profiles suggest that Zn-TPyP nanotubes are formed through an on-pathway aggregate mechanism. Additionally, the lag time for the transformation is dependent on surfactant concentrations, while the kinetics fitting to the Finke–Watzky model shows a decrease in the nucleation rate (*k*_1_) on increasing the surfactant concentration (Fig. 3b and e). Since the kinetics of growth could be related to kinetic stability of micelle formation in acid-base neutralization, therefore, we suggested that the reaction kinetics become slow due to the enhanced kinetic stability of micelle formation. In a control experiment without surfactant as well as at surfactant concentrations below 0.1 mM, immediate precipitation was observed, indicating that micelle-induced kinetic stabilization is essential for the growth of Zn-TPyP nanotubes. The results overall suggest an encapsulation-induced nucleation and elongation mechanism. From the SEM image (Fig. S2), Zn-TPyP nanoparticles in the formation of micelles were observed immediately after injection of the Zn-TPyP-H_4_^4+^ acidic aqueous solution into the basic surfactant solution. Subsequent self-assembly was driven by intermolecular axial coordination (Zn–N) and noncovalent interactions, such as hydrophobic forces and aromatic π–π stacking between molecules or surfactants, leading to the nucleation and growth of J-aggregated Zn-TPyP nanostructures. This was further supported by dynamic light scattering (DLS) measurements (Fig. S9), which showed an increase in hydrodynamic radius for the J-aggregated Zn-TPyP compared to that of the initially formed micelles, confirming the formation of larger nanostructures with a length of *ca.* 240 nm (Fig. S10). Additionally, solid-state IR spectra of micelle-encapsulated and J-aggregated Zn-TPyP were obtained (Fig. S11). The micelle-encapsulated Zn-TPyP displayed a characteristic strong band at 1598 cm^−1^, attributed to the C=N stretching vibration of the *meso*-attached pyridine groups. In contrast, the J-aggregated Zn-TPyP nanotubes exhibited two distinct peaks at 1676 and 1617 cm^−1^, indicative of Zn–N metal–ligand coordination between the meso-attached pyridine groups.^16^ This IR observation further supports the nucleation–growth process driven by axial coordination bonds.

**Fig. 4:**
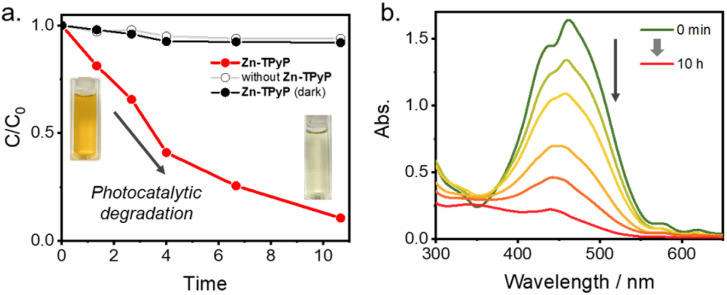
Photocatalytic degradation of methyl orange (MO) by Zn-TPyP nanotubes; (a) photocatalytic degradation efficiency (*C*/*C*_0_) as a function of irradiation time in the absence (gray line) and presence (red line) of Zn-TPyP nanotubes and dark (black line), (b) UV-vis absorption spectra of MO recorded at 2 h intervals, showing the progressive degradation in the presence of Zn-TPyP nanotubes.

The photocatalytic degradation of methyl orange (MO) in aqueous solution was employed to evaluate the photocatalytic activity of Zn-TPyP nanotubes under visible light irradiation, as shown in Fig. 4. In the absence of either visible light or Zn-TPyP nanotubes, a low degree of dye degradation was observed, indicating that both visible light irradiation and the presence of Zn-TPyP nanotubes are necessary for effective MO degradation. This highlights the crucial role of the photocatalyst and light in driving the degradation process. Time-dependent measurements of the absorption spectra of MO in aqueous solution, in the presence of Zn-TPyP nanotubes under visible light, are presented in Fig. 4b. The degradation of MO dye was monitored by measuring the decrease in absorbance at 461 nm over time. The photocatalytic efficiency of the Zn-TPyP nanotubes for degrading MO was quantified using the degradation efficiency formula ((*C*_0_ − *C*)/*C*_0_), where (*C*_0_) is the initial concentration of MO and (*C*) is the concentration at time (*t*). These results suggest that the Zn-TPyP nanotubes exhibit efficient MO degradation under visible light, and this photocatalytic activity is attributed to the intrinsic photophysical properties of the aggregated Zn-TPyP structures. The reaction kinetics for MO decomposition were investigated using the pseudo-first-order model, expressed as (ln(*C*_0_/*C*) = *kt*), which is typically applied in photocatalytic degradation experiments for low initial concentrations of the pollutant or dye.^17^ In this equation, (*k*) represents the pseudo-first-order rate constant. By plotting (ln(*C*_0_/*C*)) *versus* (*t*) using the data from Fig. 4a, we determined the rates of MO decomposition by the photocatalysts. The first-order rate constant for the degradation of MO by Zn-TPyP nanotubes was found to be 0.035 min^−1^ at 298 K (Fig. S12). Based on the photophysical properties of Zn-TPyP nanotube and our control experiments, we propose that MO degradation proceeds through a pathway mediated by reactive oxygen species (ROS).^18^ Upon visible-light excitation, Zn-TPyP nanotubes populate a triplet state, which can transfer energy to dissolved oxygen to generate singlet oxygen (1O_2_). In addition, electron transfer from the excited Zn-TPyP may produce a smaller amount of superoxide radicals (O_2_˙^−^). These ROS species subsequently react with MO by attacking the azo bond and aromatic rings, leading to progressive oxidative degradation.

We note that the high pH required to stabilize the encapsulation-induced Zn-TPyP nanotubes represents a practical limitation; however, this challenge may be addressed in future studies by designing surfactants or assembly environments that provide comparable kinetic control under more broadly applicable conditions. Despite this limitation, our findings highlight the crucial role of nanostructure in governing photocatalytic performance and demonstrate that Zn-TPyP nanotubes function as efficient photocatalysts for environmental remediation. These results provide valuable insights into the design of advanced photocatalytic systems and highlight the broader potential of kinetically controlled supramolecular assemblies in the development of functional materials.

## Conclusions

We have demonstrated the successful kinetically controlled self-assembly of Zn-TPyP into nanotubes through a surfactant-assisted micelle formation strategy. The incorporation of a chiral surfactant, characterized by alkyl chains with carboxylic acid groups, played a crucial role in stabilizing micelle formation and suppressing spontaneous self-assembly processes. This approach enabled the encapsulation-induced nucleation and growth of Zn-TPyP nanotubes, as confirmed by comprehensive spectroscopic and microscopic analyses. Our findings reveal that the kinetic stability of micelle formation plays a crucial role in delaying the transformation of micelle formation of Zn-TPyP into thermodynamically favored J-aggregated nanotubes, allowing for precise control over the self-assembly process. Furthermore, the Zn-TPyP nanotubes exhibited photocatalytic activity in the degradation of methyl orange (MO) under visible light irradiation, as evidenced by a pseudo-first-order reaction model rate constant of 0.035 min^−1^ at 298 K. Overall, this work reveals the importance of encapsulation for kinetically controlled metallo-supramolecular polymerization. The insights gained from these observations pave the way for developing metallo-supramolecular polymers with tailored functional properties.

## Conflicts of interest

There are no conflicts to declare.

## Supplementary Material

RA-015-D5RA09001F-s001

## Data Availability

The data supporting this article have been included as part of the supplementary information (SI). Supplementary information: experimental methods, synthesis procedures, structural characterization data for desired compounds, and supporting figures. See DOI: https://doi.org/10.1039/d5ra09001f.

## References

[cit1] Aida T., Meijer E. W., Stupp S. I. (2012). Science.

[cit2] Webber M. J., Appel E. A., Meijer E. W., Langer R. (2016). Nat. Mater..

[cit3] Feng X., Wang X., Redshaw C., Tang B. Z. (2023). Chem. Soc. Rev..

[cit4] Lee Ho., Park H., Ryu D. Y., Jang W.-D. (2023). Chem. Soc. Rev..

[cit5] Chappaz-Gillot C., Marek P. L., Blaive B. J., Canard G., Bürck J., Garab G., Hahn H., Javorfi T., Kelemen L., Krupke R. (2012). et al.. J. Am. Chem. Soc..

[cit6] Jin Z., Sasaki N., Kishida N., Takeuchi M., Wakayama Y., Sugiyasu K. (2023). Chem. – Eur. J..

[cit7] Sorrenti A., Leira-Iglesias J., Markvoort A. J., de Greef T. F. A., Hermans T. M. (2017). Chem. Soc. Rev..

[cit8] Su L., Mosquera J., Mabesoone M. F. J., Schoenmakers S. M. C., Muller C., Vleugels M. E. J., Dhiman S., Wijker S., Palmans A. R. A., Meijer E. W. (2022). Science.

[cit9] Dhiman S., Sarkar A., George S. J. (2018). RSC Adv..

[cit10] Liu Y., Wang H., Li S., Chen C., Xu L., Huang P., Liu F., Su Y., Qi M., Yu C., Zhou Y. (2020). Nat. Commun..

[cit11] Wei W., Bai F., Fan H. (2019). iScience.

[cit12] Morisue M., Hoshino Y., Shimizu K., Shimizu M., Kuroda Y. (2015). Chem. Sci..

[cit13] Bai F., Sun Z., Wu H., Haddad R. E., Coker E. N., Huang J. Y., Rodriguez M. A., Fan H. (2011). Nano Lett..

[cit14] kada S., Segawa H. (2003). J. Am. Chem. Soc..

[cit15] Morris A. M., Watzky M. A., Agar J. N., Finke R. G. (2008). Biochemistry.

[cit16] Sun W., Wang H., Qi D., Wang L., Wang K., Kan J., Li W., Chen Y., Jiang J. (2012). CrystEngComm.

[cit17] Shee N. K., Kim H.-J. (2022). ACS Omega.

[cit18] Schweitzer C., Schmidt R. (2003). Chem. Rev..

