# Supplementary material for: Kinetically controlled self-assembly of Zn-porphyrin nanostructures via surfactant-assisted micelle formation
Source: RSC Adv. 2025 Dec 9;15(57):48757–61. doi: 10.1039/d5ra09001f (PMC12686873; doi:10.1039/d5ra09001f)
Supplement: RA-015-D5RA09001F-s001 [file RA-015-D5RA09001F-s001.pdf]

Supplementary Information (SI) for RSC Advances.
This journal is © The Royal Society of Chemistry 2025

Electronic Supplementary Information (ESI)

Kinetically controlled self-assembly of Zn-porphyrin nanostructures via surfactant-assisted micelle formation

Yun Mi Park,[a] Ka Young Kim,[b] Juyeong Kim,[a,c] Ji Ha Lee,*[d] Jong Hwa Jung,*[a,c] Sung Ho Jung* [a,c]

[a] Department of Chemistry, Gyeongsang National University, Jinju 52828, Republic of Korea. E-mail: shjung@gnu.ac.kr

[b] Technical Support Center for Chemical Industry, Korean Research Institute of Chemical Technology (KRICT), 45 Jongga-ro, Jung-gu, Ulsan 44412, Republic of Korea.

[c] Institute of Advanced Chemistry, Gyeongsang National University, Jinju 52828, Republic of Korea.

[d] Institute for Fiber Engineering and Science (IFES), Interdisciplinary Cluster for Cutting Edge Research (ICCER), Shinshu University, Ueda 386-8567, Japan.

Table contents

1. **Experimental Methods** **S3**
2. **Synthesis and Characterization** **S4**
3. **Supporting Data (Fig. S1-S12)** **S5**
4. **Analytic Data** **S17**
5. **Supplementary References** **S22**

1. Experimental Methods

1.1 General. High-resolution fast atom bombardment (FAB) mass spectra were obtained using a JEOL JMS-700 mass spectrometer. The nuclear magnetic resonance (NMR) spectra were recorded on Bruker 300 and 500 spectrometers (300 MHz). The Fourier transform infrared (FT-IR) spectra were measured with a Jasco 4x spectrometer. Circular dichroism (CD) spectra and circularly polarized luminescence (CPL) spectra were recorded by using a JASCO J-810 spectropolarimeter and a JASCO CPL-300 spectrofluoropolarimeter in quartzose cells, respectively. The UV-visible (UV-vis) absorption spectra were recorded Jasco V-730 spectrophotometer. The field emission scanning electron microscopy (FE-SEM) images were obtained using a TESCAN S8000 instrument. The Cs-corrected transmission electron microscopy (TEM) images were obtained using a JEOL JEM-ARM200F (NEOARM) instrument. Dynamic light scattering (DLS) measurements were carried out on a Malvern Zetasizer Nano ZS instrument.

1.1 Preparation of Zn-TPyP nanoroads

Zn-TPyP (50 μM) was dissolved in HCl (0.1M) solution, stirring for 1 min. Then, 0.3mL **Zn-TPyP** solution was immediately injected into 2.7 mL of aqueous solution containing surfactant (900 μM) and NaOH (0.05M) under continuous stirring. The solution was stirred continuously for 48 hours at room temperature (25 °C). Finally, the solution was centrifuged at 4000 rpm to collect the porphyrin nanostructures and washed with Millipore water to remove free surfactants.

1.2 Calculation of Rate Constant

Data were obtained by converting to α_{agg} values with absorption obtained by time-dependent UV-Vis spectra measurements at 298K. The rate constants k_1 and k_2 for the aggregation process following a cooperative mechanism were obtained by fitting the previously described data to the F-W model.[1,2]

$$A \xrightarrow{k_1} B$$
$$A+B \xrightarrow{k_2} 2B$$

$$[B]_t = [A]_0 - \frac{\frac{k_1}{k_2} + [A]_0}{1 + \frac{k_1}{k_2[A]_0} exp(k_1 + k_2[A]_0)t}$$

Here $[A]_0$ is initial concentration of monomeric state, $[B]_t$ is concentration of polymeric state at time t and k_1 and k_2 correspond to average rate constants for nucleation and elongation steps.

2. Synthesis and characterization

Unless otherwise noted, chemical reagents and solvents were purchased from commercial suppliers (Tokyo Chemical Industry (TCI), Sigma Aldrich) and used without further purification.

2.1 Synthesis of surfactant

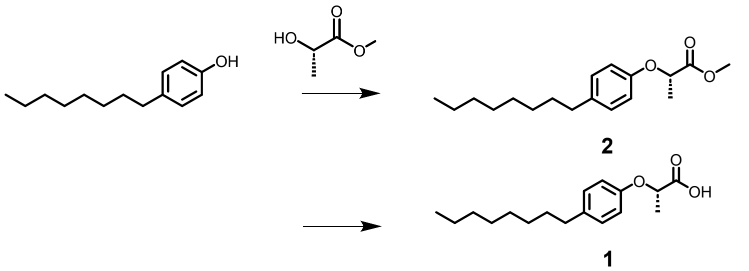

Scheme S1. Synthesis of surfactant **1**

Synthesis of compound 2

4-Octylphenol (0.6768g, 3.28mmol), methyl (L)-lactate (0.376mL, 3.94mmol) and triphenylphosphine (1.033g, 3.94mmol) were dissolved in dry THF (30mL) with stirring at under an argon atmosphere. To this mixture, a solution of diethyl azodicarboxylate (DEAD) (0.7178mL, 3.94mmol) in dry tetrahydrofuran (THF) (5mL) was added dropwise over a period of 30 minutes at 273K, and the mixture was warmed up to room temperature with stirring overnight. The THF was removed in vacuo, and the mixture was extracted with dichloromethane (DCM) and water. The solid material was purified through column chromatography (SiO_2; Hex/DCM 1:2) (Yield: 73%) ^{1}H NMR ($CDCl_3$, 300MHz) δ7.00-6.95 (d, 2H, Ar-H), δ6.72-6.67 (d, 2H, Ar-H), δ4.67-4.60 (q, 1H, -C*H*(-CH_3)-COOH), δ3.65 (s, 3H, -COO-CH_3) δ2.46-2.41 (t, 2H, -C-CH_2-(C_7H_{15})), δ1.52-1.43 (d, 3H, -CH(-CH_3)-COO-CH_3), δ1.23-1.16 (m, 2H, -C-CH_2-CH_2-(C_6H_{13})), δ0.81-0.77 (t, 3H, - (C_7H_{14})-CH_3).

Synthesis of surfactant 1

A solution of compound **2** (0.15g, 0.513mmol) in THF/MeOH (3mL/1mL) was treated with NaOH (0.0408g, 1.0M, 1.02mmol) and stirred at room temperature. The reaction mixture was acidified to pH 1 using 1M HCl and extracted with ethyl acetate (EtOAc) and water three times. The product was purified by recrystallization using DCM/EtOAc. (Yield: 97%) FT-IR (KBr, cm^{-1}): 3456 (COOH), 1735 (C=O), 1250 (C–O–C). ^{1}H NMR ($CDCl_3$, 300MHz) δ11.3 (s, 1H, -COO*H*), δ7.17-7.14 (d, 2H, Ar-H), δ6.91-6.87 (d, 2H, Ar-H), δ4.85-4.78 (q, 1H, -C*H*(-CH_3)-COOH), δ2.65-2.60 (t, 2H, -C-CH_2-(C7H15)), δ1.72-1.68 (d, 3H, -CH(-CH_3)-COOH), δ1.67-1.62 (m, 2H, -C-CH_2-CH_2-(C_6H_{13})), δ1.51-1.36 (m, 10H, Alkyl chain), δ1.01-0.97 (t, 3H, -(C7H14)-C*H*3). ^{13}C NMR ($CDCl_3$, 75MHz) δ(ppm) 177.6, 155.2, 136.4, 129.4, 115.0, 72.3, 35.0, 31.9, 31.6, 29.8, 29.7, 29.4, 29.3, 29.2, 22.6, 18.4, 14.1. MS-FAB$^+$ for surfactant **1** : calculated for $[C_{17}H_{26}O_3]^+$: *m*/*z* = 278.18 $[M]^+$; found: 278.22 $[M]^+$.

3. Supporting Data (Fig. S1-S12)

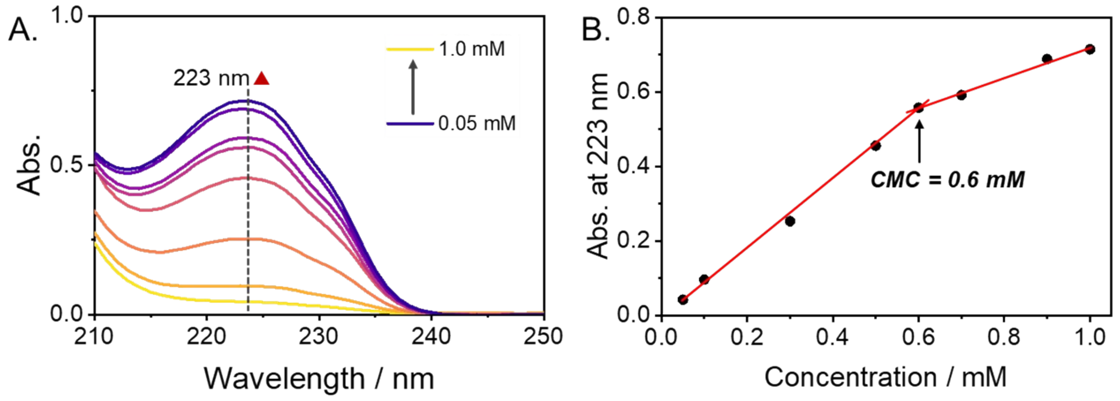

Fig. S1 (A) UV–vis absorption spectral changes of surfactant at varying surfactant concentrations. (B) Plot of absorption at 223 nm as a function of surfactant concentration.

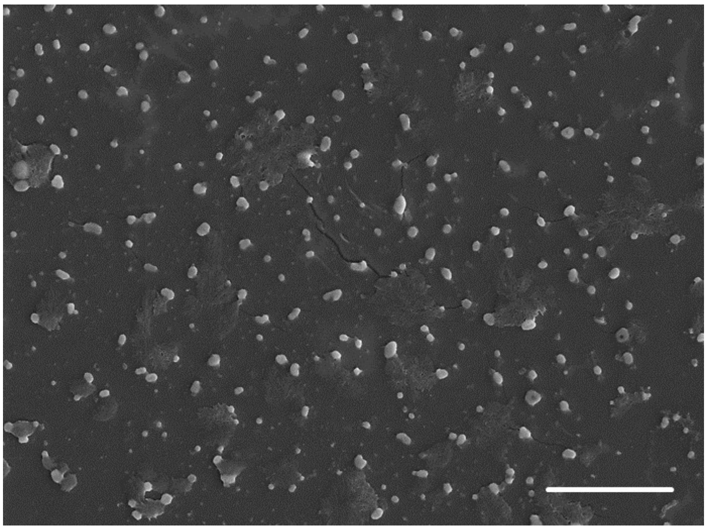

Fig. S2 SEM image of micelle formation of **Zn-TPyP**. Scale bar = 10 μm.

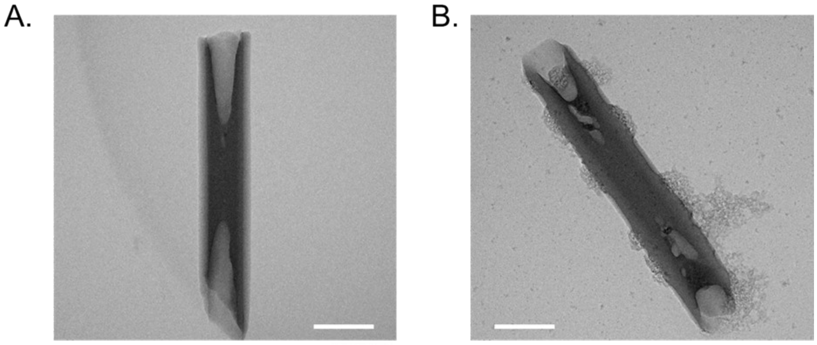

Fig. S3 (A-B) TEM images of nanotube formation of **Zn-TPyP**. Scale bar = 50 nm.

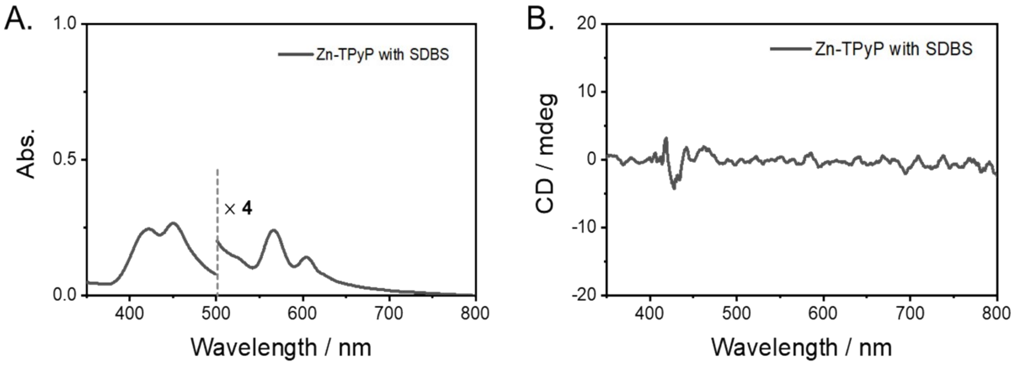

Fig. S4 (A) UV-Vis and (B) CD spectrum of **Zn-TPyP** (50 μM) with SDBS (1.0 mM).

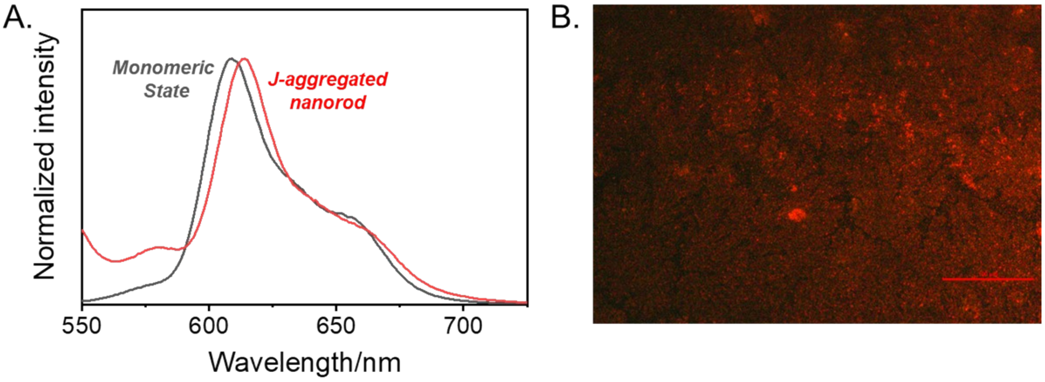

Fig. S5 (A) Fluorescence spectra of monomeric **Zn-TPyP** (grey line) and *J*-aggregated **Zn-TPyP** nanotube (red line). (B) Fluorescence microscopy image of *J*-aggregated **Zn-TPyP** nanotube (scale bar = 100 μm).

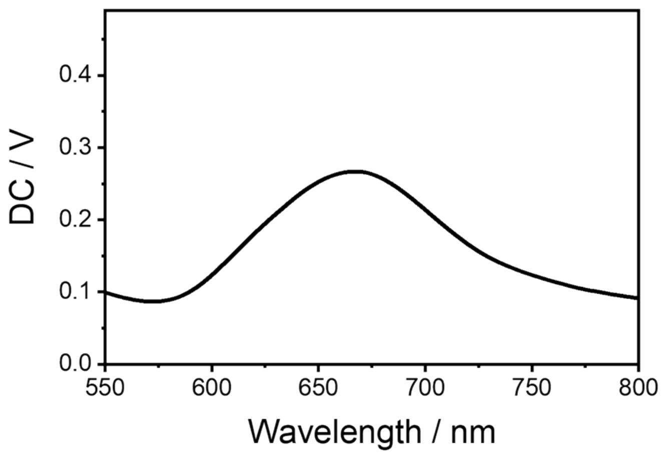

Fig. S6 Total luminescence (DC) spectra of **Zn-TPyP** nanotube (corresponding to Figure 2b).

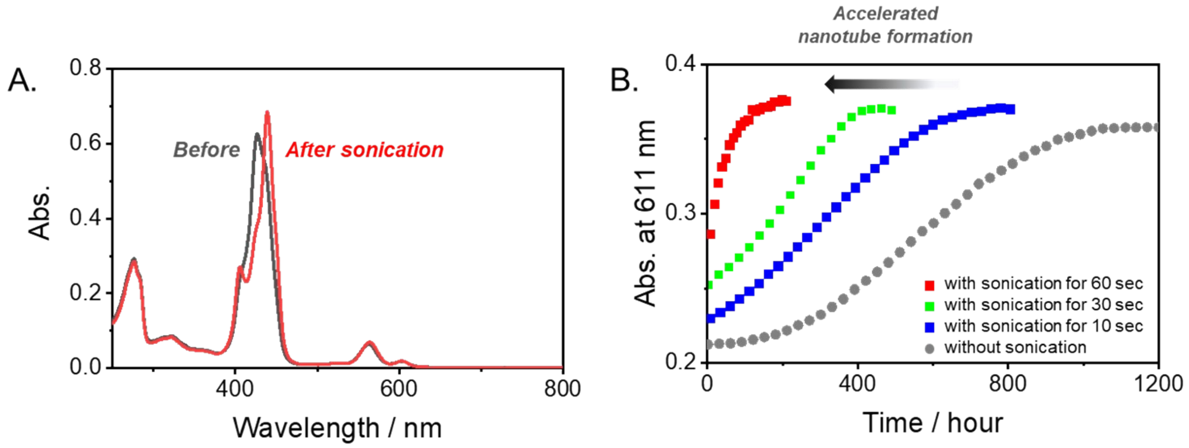

Fig. S7 (A) UV–vis absorption spectra of **Zn-TPyP** before and after sonication (60 sec). (B) Time-dependent absorption changes of **Zn-TPyP** monitored at 611 nm, showing accelerated nanotube formation upon applying sonication for 10-60 sec.

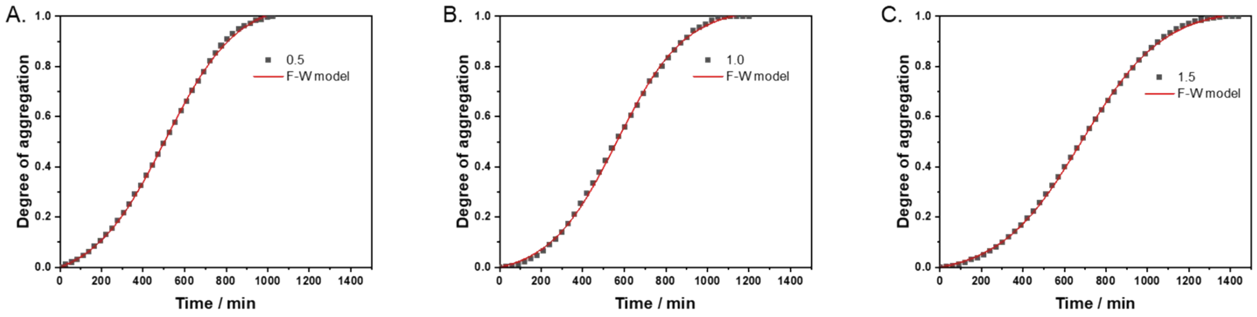

Fig. S8 (A-C) Time-dependent degree of aggregation of **Zn-TPyP** (50 μM) at different surfactant concentrations: (A) 0.5 mM, (B) 1.0 mM, and (C) 1.5 mM.

Table S1. Rate constants and corresponding R^2 values obtained by fitting the kinetic curves in Figure S8 using the Finke–Watzky (F–W) model.

Concentration (mM)	K_1 (min^{-1})	K_2 ($\mu M^{-1} min^{-1}$)	R^2
0.5	2.97×10^{-4}	5.72×10^{-3}	0.999
1.0	1.88×10^{-4}	5.10×10^{-3}	0.999
1.5	1.54×10^{-4}	4.76×10^{-3}	0.999

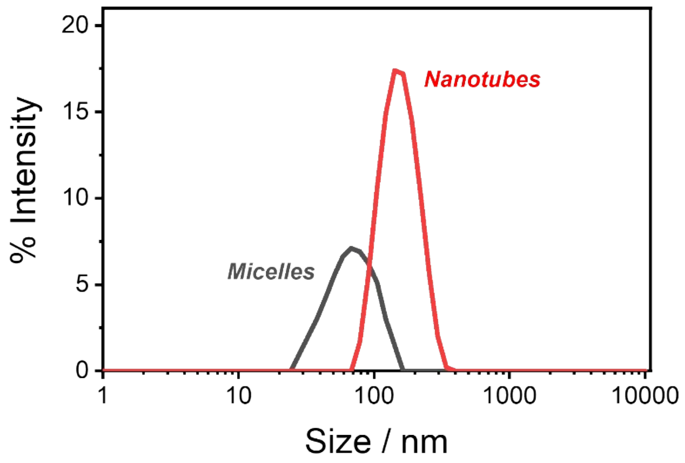

Fig. S9 DLS size distribution profiles of **Zn-TPyP** micelles and nanotubes.

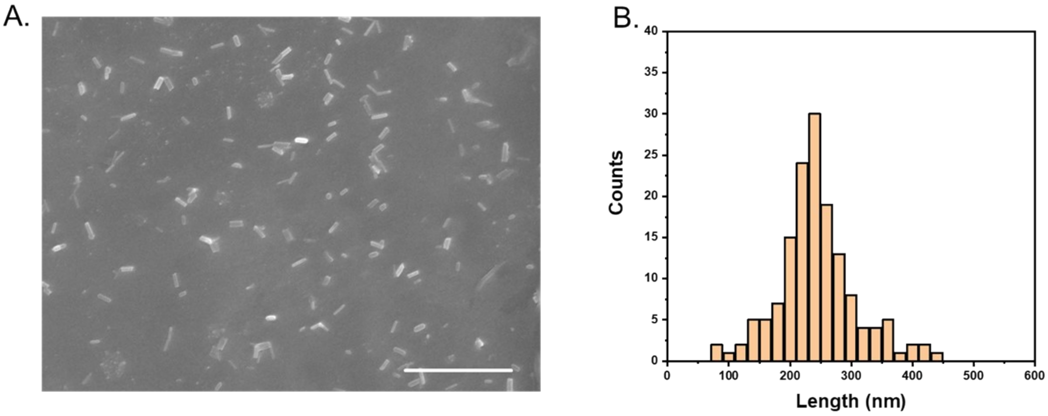

Fig. S10 (A) SEM image and (B) length distribution of **Zn-TPyP** nanotubes. Scale bar = 2.0 μm.

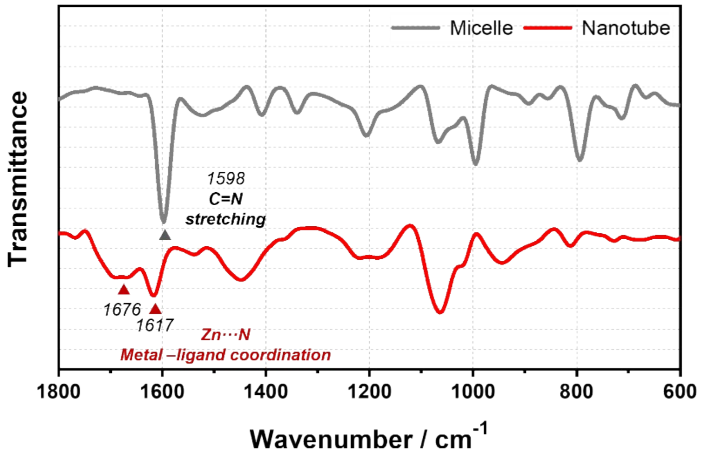

Fig. S11 IR spectra of **Zn-TPyP** in micelle and nanotube states.

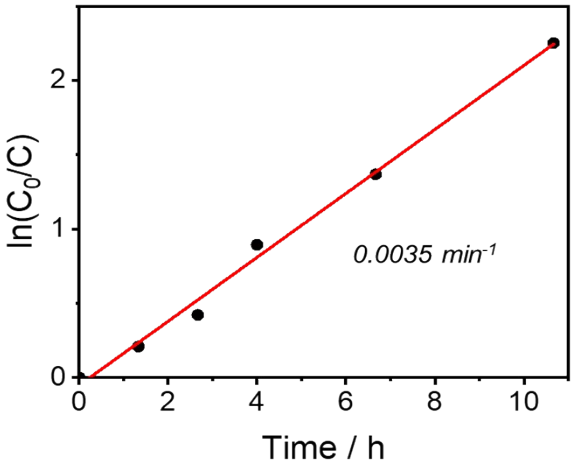

Fig. S12 Pseudo-first-order kinetic plot for the photocatalytic degradation of MO by **Zn-TPyP** nanotubes.

4. Analytic Data

^{1}H-NMR and ^{13}C-NMR spectrum

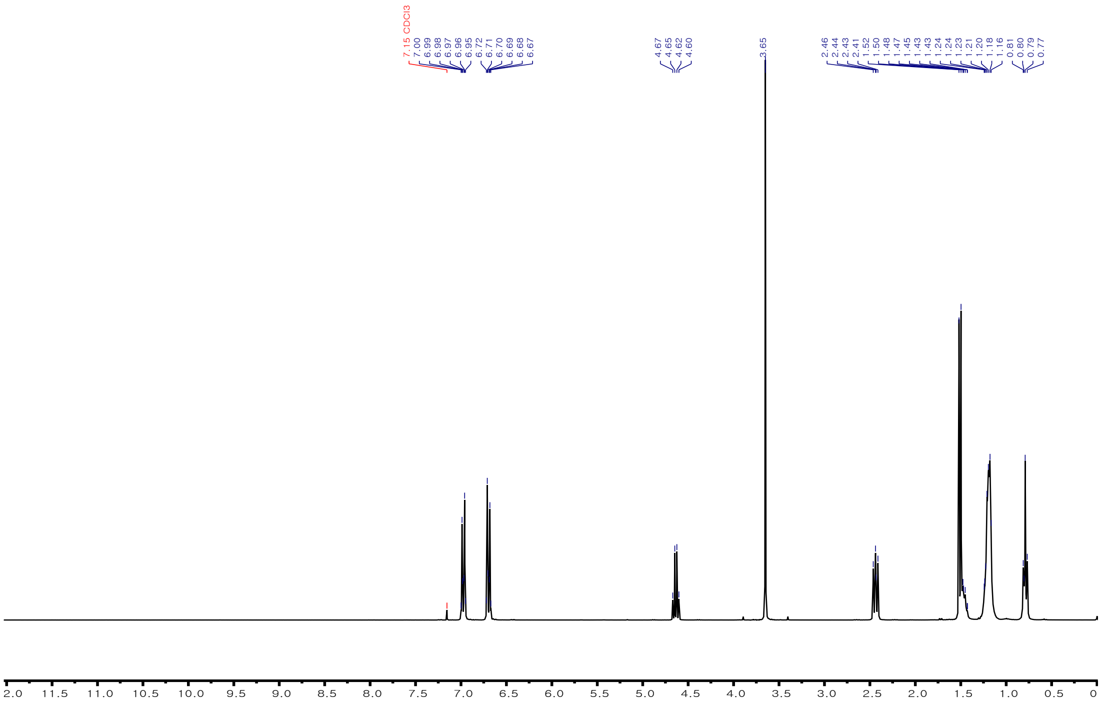

^{1}H-NMR spectrum of Compound **2**

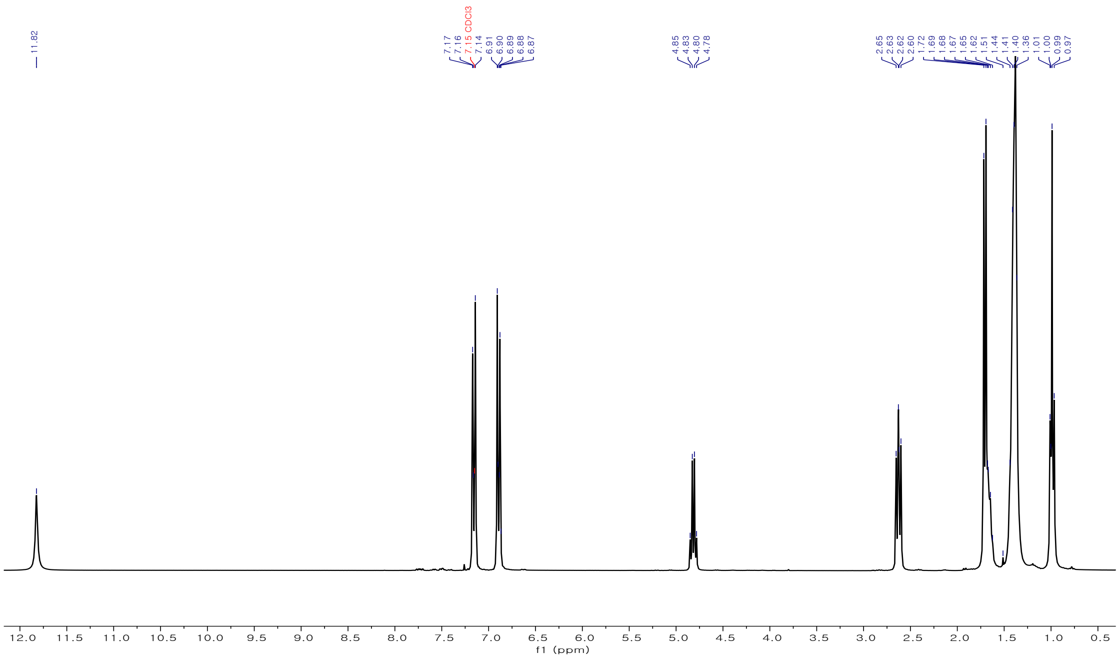

^{1}H-NMR spectrum of Compound **1**

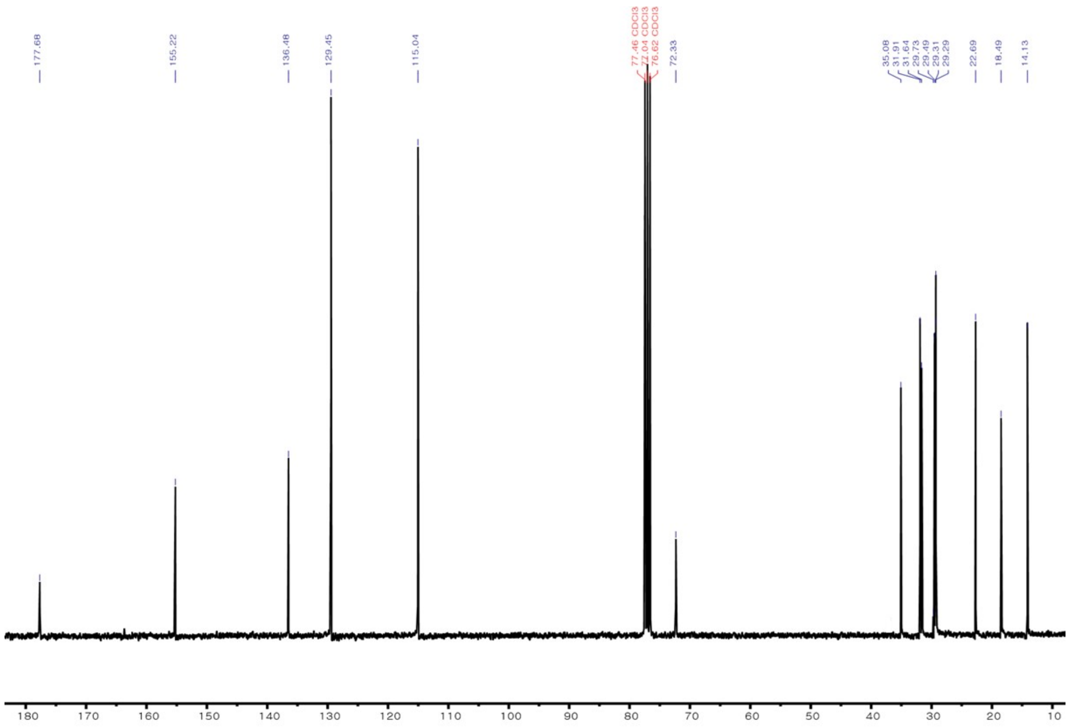

^{13}C-NMR spectrum of Compound **1**

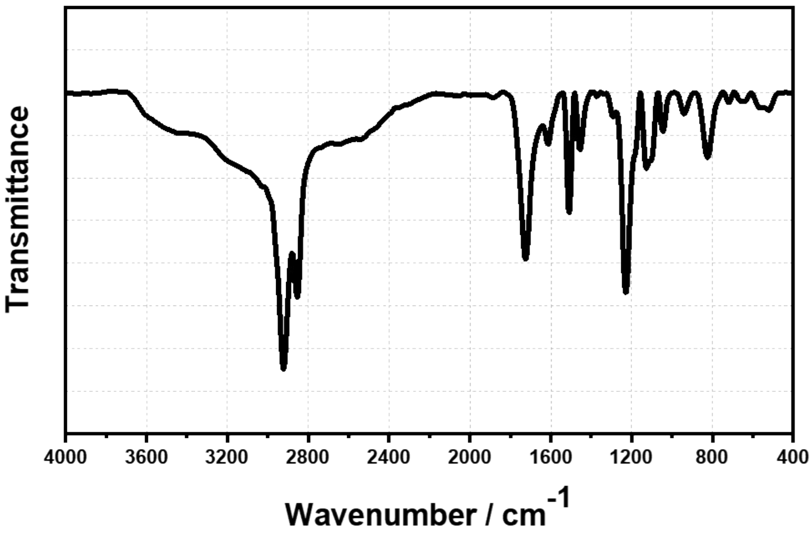

FT-IR spectrum of Compound **1**

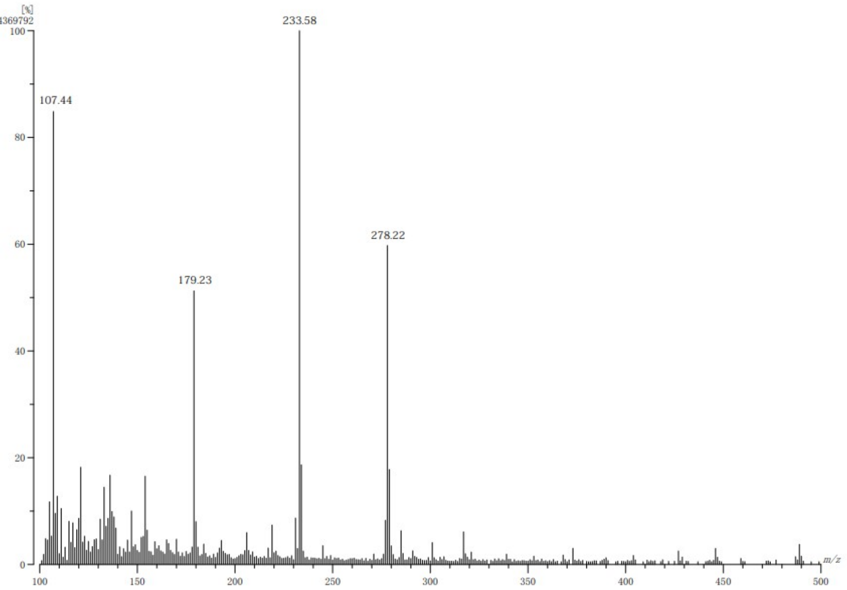

Mass spectrum of Compound **1**

5. Supplementary References

[1] L. Bentea, M. A. Watzky, R. G. Finke, *J. Phys. Chem. C* **2017**, *121*, 5302-5312.

[2] A. M. Morris, R.G. Finke, *Biophys. Chem.* **2009**, *9*, 9-15.
